# Linkage mapping identifies a non-synonymous mutation in *FLOWERINGLOCUST* (*FT-B1*) increasing spikelet number per spike

**DOI:** 10.1038/s41598-020-80473-0

**Published:** 2021-01-15

**Authors:** Jonathan Brassac, Quddoos H. Muqaddasi, Jörg Plieske, Martin W. Ganal, Marion S. Röder

**Affiliations:** 1 Leibniz Institute of Plant Genetics and Crop Plant Research (IPK), Corrensstr 3, 06466 Stadt Seeland OT Gatersleben, Germany; 2 TraitGenetics GmbH, Am Schwabeplan 1b, 06466 Stadt Seeland OT Gatersleben, Germany; 3 Present Address: European Wheat Breeding Center, BASF Agricultural Solutions GmbH, Am Schwabeplan 8, 06466 Stadt Seeland OT Gatersleben, Germany

**Keywords:** Agricultural genetics, Plant breeding, Plant genetics

## Abstract

Total spikelet number per spike (TSN) is a major component of spike architecture in wheat (*Triticum* *aestivum* L.). A major and consistent quantitative trait locus (QTL) was discovered for TSN in a doubled haploid spring wheat population grown in the field over 4 years. The QTL on chromosome 7B explained up to 20.5% of phenotypic variance. In its physical interval (7B: 6.37–21.67 Mb), the gene *FLOWERING* *LOCUS* *T* (*FT-B1*) emerged as candidate for the observed effect. In one of the parental lines, *FT-B1* carried a non-synonymous substitution on position 19 of the coding sequence. This mutation modifying an aspartic acid (D) into a histidine (H) occurred in a highly conserved position. The mutation was observed with a frequency of ca. 68% in a set of 135 hexaploid wheat varieties and landraces, while it was not found in other plant species. *FT-B1* only showed a minor effect on heading and flowering time (FT) which were dominated by a major QTL on chromosome 5A caused by segregation of the vernalization gene *VRN-A1*. Individuals carrying the *FT-B1* allele with amino acid histidine had, on average, a higher number of spikelets (15.1) than individuals with the aspartic acid allele (14.3) independent of their *VRN-A1* allele. We show that the effect of TSN is not mainly related to flowering time; however, the duration of pre-anthesis phases may play a major role.

## Introduction

In light of a growing world population, the increase of wheat yield and the genetic mechanisms behind are of immediate interest. It was shown that grain yield in German winter wheat cultivars was highly correlated with grain number per spike^1^ and, therefore, the genes and mechanisms determining inflorescence and spike architecture in cereals have been in the focus of research (reviewed in Gauley and Boden^2^; Koppolu and Schnurbusch^3^). The unbranched spike of wheat contains one spikelet per node and is terminated with a single terminal spikelet at the apical end of the inflorescence. Each spikelet contains 8–12 florets and produces usually 3–5 grains per spikelet^1^. Important determinants of grain number are the floret fertility^4^ as well as spike fruiting efficiency (grains per unit spike dry weight at anthesis)^5,6^. Among the complex network of spike-related traits, total spikelet number per spike (TSN) was studied in several reports. In genetic approaches, TSN appears usually as a quantitative trait and several quantitative trait loci (QTL) were described^7–9^. A few genes influencing TSN are known; among them are (1) the *Q* gene involved in wheat domestication^10,11^, (2) a putative ortholog to rice *MOC1*^12^, and (3) a wheat ortholog (*TaAPO-A1* or *WAPO-A1*) to rice *ABERRANT* *PANICLE* *ORGANIZATION* (*APO1*)^13–15^.

Besides the modulation of spikelet meristem identity genes, the genetic regulation of spikelet initiation through timing and florigenic signals plays a major role in the determination of TSN. Homologs of *Arabidopsis* *FLOWERING* *LOCUS* *T* (*FT*)^16–18^, such as *HEADING* *DATE* *3a* (*Hd3a*) and *RICE* *FLOWERING* *LOCUS* *T* *1* (*RFT1*) in rice promote transition to flowering and tillering^19–21^. In wheat, the *FT-B1* gene on chromosome 7B was originally reported as *VRN-B3* that affects the vernalization requirement in wheat and—besides the three *VRN-1* genes—that determines the spring or winter growth type of hexaploid wheat^22,23^. In wheat *FT-B1* acts downstream of *PHOTOPERIOD-1* (*PPD-1*) gene to influence spikelet number, with loss of *FT-B1* resulting in increased spikelet number in a thermally responsive manner^2,24,25^. An ortholog of the maize domestication gene *TEOSINTE* *BRANCHED* *1* (*TB1*) was reported to interact with *FT-B1* in a dosage-dependent manner to promote the formation of paired spikelets in wheat by activating a cascade of floral meristem identity genes^26^.

*FT-B1* is a member of an evolutionarily well-conserved gene family [phosphatidylethanolamine-binding protein (PEBP)] that is present in all taxa including bacteria, animals, and plants^27^. Among angiosperms, three subfamilies are described *FT*-like, *MFT*-like (*MOTHER* *OF* *FT* *AND* *TFL*), and *TFL1*-like genes (*TERMINAL* *FLOWER1*)^17^. Phylogenetic analyses identified *MFT*—the only member of the PEBP family in mosses—as the ancestral gene, and its duplication in *FT*/*TFL1*-like genes and their divergence coincided with the evolution of vascular plants^28,29^. Although sharing a high homology, the genes have been described to be partially redundant as flowering promoters (*FT* and *MFT*) or to act in an antagonist manner (*TFL1*)^17,30^. Compared to the two other gene families, *FT*-like genes, evolving via small- and large-scale duplications, are the most numerous among monocots with 13 paralogs identified in rice, five in barley, and nine in wheat based on expressed sequences tags (ESTs) data^31,32^. Due to their impact on the phenology and flowering architecture of crops, *FT*-like genes have been a target of selection during domestication and subsequent range expansion to adapt to new environments^33,34^.

In this study, we performed linkage mapping to investigate the genetic basis of total spikelet number per spike and its associated traits viz., spike length, heading date, and flowering time in a doubled haploid spring wheat population. Our analyses identified a large-effect locus that harbored *FT-B1*—a candidate gene for TSN. The origin and frequency of a non-synonymous mutation associated with an increased spikelet number was investigated by analyzing a large diversity of land plants and a panel of hexaploid wheat varieties. Moreover, we also investigated the interaction of *FT-B1* with the vernalization gene *VRN-A1* that segregated in the studied population.

## Results

### Description of phenotypic data

**Figure 1:**
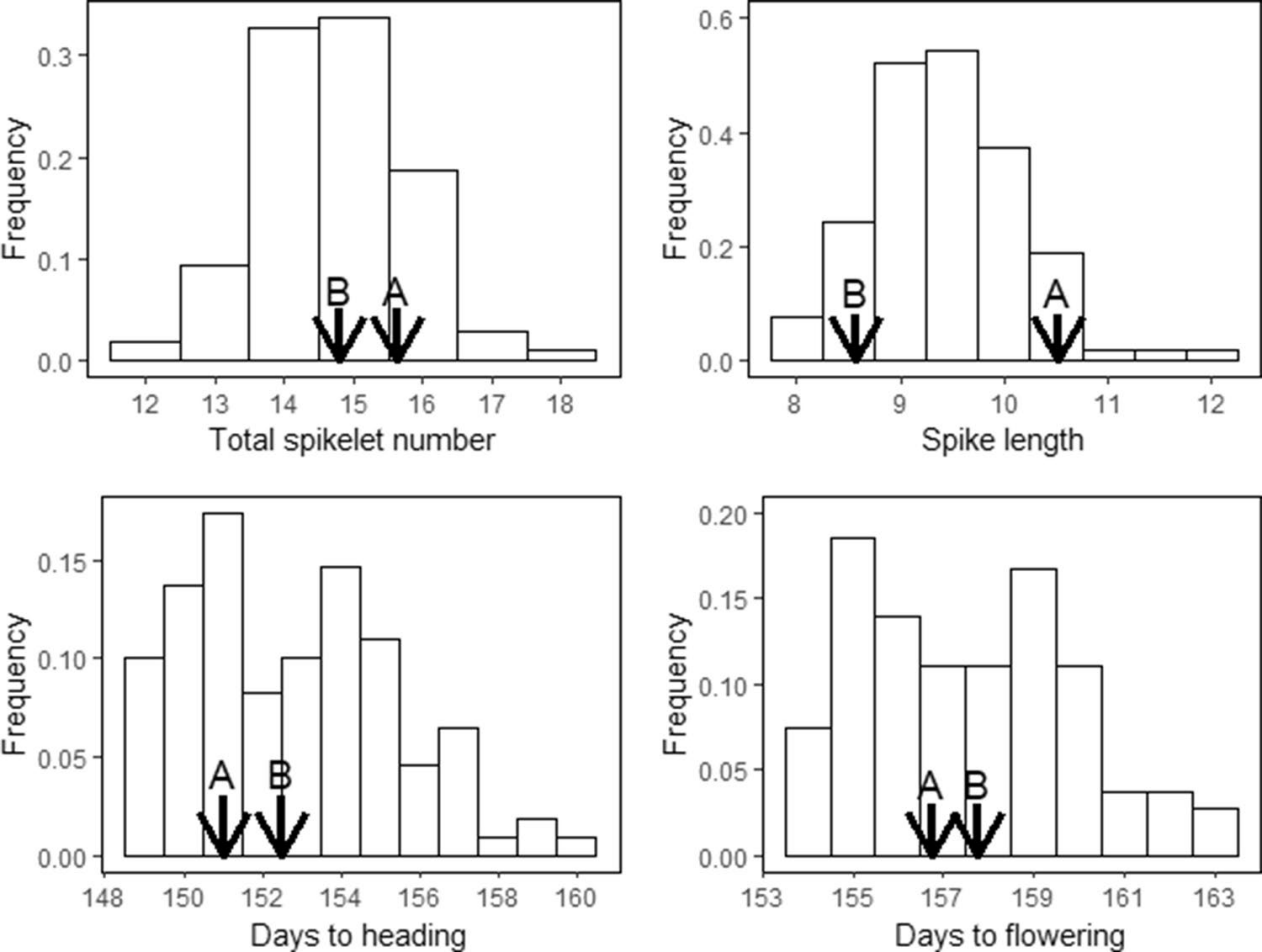
Phenotypic distribution of the best linear unbiased estimations (BLUEs) of the investigated traits. Arrows indicate the phenotypes of the parental lines with parent A corresponding to TRI-10703 and parent B to TRI-5310.

**Figure 2:**
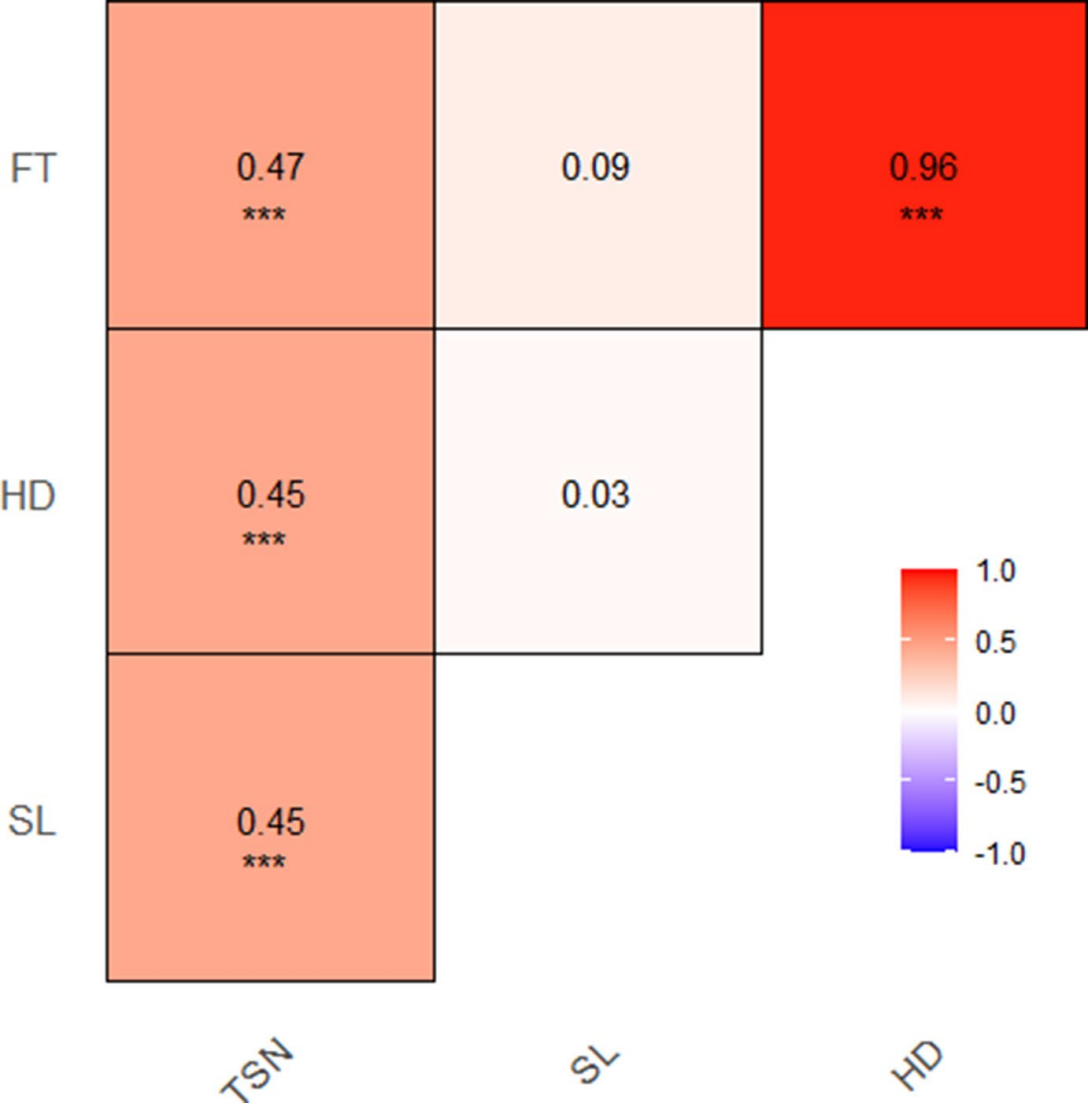
Pearson’s correlation coefficient of the best linear unbiased estimations (BLUEs) of total spikelet number (TSN), spike length (SL), heading date (HD), and flowering time (FT). Significance of the correlations is indicated with ***P < 0.001.

The doubled haploid (DH) population-2 described in Muqaddasi et al.^35^ was grown on the field and characterized for 4 years (2016–2019) for total spikelet number (TSN) per spike, spike length (SL), heading date (HD), and flowering time (FT). The best linear unbiased estimations (BLUEs) showed a wide variation within and across years for all traits, with TSN and SL being normally distributed (Shapiro–Wilk test: *P* = 0.82 for TSN, *P* = 0.06 for SL, *P* = 0.001 for HD, and *P* = 0.003 for FT). Although the parental lines did not differ much for their flowering traits (HD and FT), the population presented a bimodal distribution (Fig. 1) resulting from the segregation of the vernalization genes *VRN*-*A1* and *VRN*-*B1*^35^. The parental line TRI-10703 carried the dominant allele *Vrn-A1a* and the recessive allele *vrn-B1*, while TRI-5310 harbored the recessive allele *vrn-A1* and the dominant allele *Vrn-B1*. For the spike morphology traits (TSN and SL), the parental genotype TRI-10703 (parent-A) systematically performed better than parental genotype TRI-5310 (parent-B; Fig. 1, Supplementary Figure S1). The ANOVA indicated significant (*P* < 0.001) genotypic and environmental differences for all traits. High repeatability values (*H*^2^ = 0.89–0.96) were obtained for biological replicates of TSN and SL. Broad sense heritability values calculated over 4 years ranged between 0.71 for SL and 0.88 for FT and HD (Table 1). The Pearson correlation coefficient calculated based on BLUEs indicated a positive and significant (*P* < 0.001) correlation of TSN with SL, HD and FT, and between HD and FT, while the correlation between SL and the flowering traits was null (Fig. 2).

**Table 1:** Summary statistics for total spikelet number (TSN), spike length (SL), heading date (HD), and flowering time (FT) across 4 years and their corresponding best linear unbiased estimations (BLUEs).

Trait	Parameter	2016	2017	2018	2019	BLUEs
TSN	Minimum	8.10	13.40	12.80	12.10	12.25
	Mean	12.85	16.16	14.99	14.90	14.70
	Maximum	17.50	19.20	17.80	18.10	17.57
	σ^2^_*G*_	1.99	1.36	1.00	1.50	0.84
	σ^2^_*GxE*_	–	–	–	–	0.60
	σ^2^_*e*_	2.07	1.02	0.96	1.34	1.77
	*H*^2^	0.91	0.93	0.91	0.92	0.81
SL	Minimum	5.00	8.05	7.55	7.50	7.90
	Mean	8.30	10.70	9.11	9.78	9.44
	Maximum	10.90	14.00	12.15	12.40	12.18
	σ^2^_*G*_	0.86	1.39	0.66	0.74	0.37
	σ^2^_*GxE*_	–	–	–	–	0.47
	σ^2^_*e*_	0.99	0.74	0.35	0.84	1.17
	*H*^2^	0.90	0.95	0.95	0.90	0.71
HD	Minimum	153	148	144	149	148.93
	Mean	157.04	151.30	151.56	150.91	152.80
	Maximum	165	159	168	156	159.60
	σ^2^_*G*_	–	–	–	–	6.10
	σ^2^_*GxE*_	–	–	–	–	–
	σ^2^_*e*_	–	–	–	–	3.28
	*H*^2^	–	–	–	–	0.88
FT	Minimum	157	152	150	154	153.75
	Mean	160.99	157.51	155.91	156.28	157.69
	Maximum	171	163	170	160	163.5
	σ^2^_*G*_	–	–	–	–	5.01
	σ^2^_*GxE*_	–	–	–	–	–
	σ^2^_*e*_	–	–	–	–	2.79
	*H*^2^	–	–	–	–	0.88

*σ*^*2*^_*G*_ genotypic variance, *σ*^*2*^_*GxE*_ genotype-by-environment variance, *σ*^*2*^_*e*_ residual variance, *H*^*2*^ repeatability among replicates (single years) or broad-sense heritability (BLUEs).

### Mapping of quantitative trait loci (QTL)

**Figure 3:**
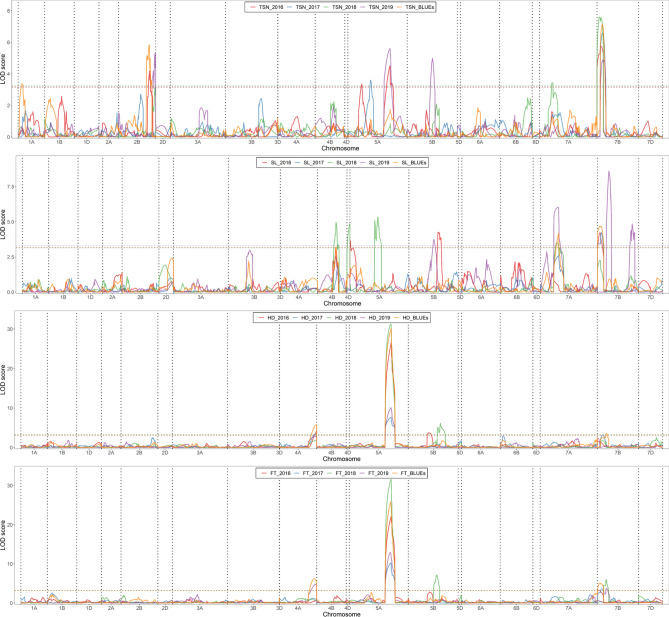
Quantitative trait loci (QTL) map of total spikelet number (TSN), spike length (SL), heading date (HD), and flowering time (FT) in a DH-population of spring wheat grown over 4 years and their corresponding best linear unbiased estimations (BLUEs). The horizontal dashed lines indicate the corresponding logarithm of odds (LOD) threshold estimated at α = 0.05 with 1000 permutations to assess the QTL significance.

Composite interval mapping identified a total of 56 individual QTL at 28 loci for all years, all traits and their resulting BLUEs (Table 2, Supplementary Table S1). However, only a single major QTL per trait was found consistent, i.e., present in all the years and their corresponding BLUEs, on chromosome 5A for FT and HD and on chromosome 7B for TSN (Fig. 3, Table 2). No consistent QTL were identified for SL. The FT (*QFt.ipk-5A*) and HD (*QHd.ipk-5A)* QTL collocated at 145.01–171.23 cM and explained 29.7–66.4% and 23–63.6% of phenotypic variance, respectively. These QTL most probably correspond to the vernalization gene *VRN-A1* (*TraesCS5A02G391700*). For *QTsn.ipk-7B—*located at 0–33.46 cM—the phenotypic variance amounted to 13.1–20.5%. Interestingly, minor QTL for the three other traits (viz., *QHd.ipk-7B*, *QFt.ipk-7B.1*, *QFt.ipk-7B.2*, and *QSl.ipk-7B.1*) were identified within or overlapping the *QTsn.ipk-7B* interval.

**Table 2:** Summary of quantitative trait loci (QTL) for total spikelet number (TSN), spike length (SL), heading date (HD), and flowering time (FT) across 4 years and their corresponding best linear unbiased estimations (BLUEs).

QTL	Environment	LOD score	LOD interval (cM)	*R*^*2*^ (%)	Additive effect
*QTsn.ipk-1A*	BLUEs	3.36	5.19–23.32	7	− 0.27
*QTsn.ipk-2B.1*	2016/BLUEs	4.2–5.84	111.32–131.49	10.2–12.8	− 0.5 to − 0.39
*QTsn.ipk-2B.2*	2019	5.28	141.81–148.22	13.2	− 0.49
*QTsn.ipk-5A.1*	2016/2017	3.36–3.6	43.2–99.98	8.2–9.1	− 0.38 to 0.47
*QTsn.ipk-5A.2*	2016/2019	4.51–5.61	145.01–177.1	11–14.2	− 0.56 to − 0.54
*QTsn.ipk-5B*	2019	4.98	88.84–101.21	12.4	0.51
*QTsn.ipk-7A*	2018	3.43	34.57–54.46	8.6	0.31
*QTsn.ipk-7B*	*ALL*	4.87–7.59	0–33.46	13.1–20.5	0.44 to 0.59
*QSl.ipk-4B*	2016/2018/BLUEs	3.2–4.96	62.01–82.01	7.4–11.6	0.20 to 0.29
*QSl.ipk-4D*	2018	4.85	0–13.66	12	0.29
*QSl.ipk-5A.1*	2016	3.76	0–4.45	10	0.32
*QSl.ipk-5A.2*	2018	5.34	106.39–118.65	12.7	0.3
*QSl.ipk-5B.1*	2016	4.26	113.75–129.91	11.7	− 0.37
*QSl.ipk-5B.2*	2019	3.74	88.84–111.19	7.8	0.27
*QSl.ipk-7A*	2018/2019/BLUEs	3.34–6.02	54.46–75.24	9.9–16.3	0.24 to 0.40
*QSl.ipk-7B.1*	2016/2017/BLUEs	4.2–4.67	0–13.31	11.1–15	0.25 to 0.47
*QSl.ipk-7B.2*	2019	8.61	37.92–50.71	23.8	0.45
*QSl.ipk-7B.3*	2019	4.85	127.74–148.26	12.3	− 0.33
*QHd.ipk-4A*	2016/2017/BLUEs	3.5–5.65	115.64–149.36	5.1–10	0.55 to 0.86
*QHd.ipk-5A*	*ALL*	7.65–30.18	145.01–171.23	23–63.6	− 4.48 to − 0.83
*QHd.ipk-5B.1*	2016	3.73	75.44–88.84	4.9	0.56
*QHd.ipk-5B.2*	2018	6.1	126.06–138.28	6.4	1.53
*QHd.ipk-7B*	2017/BLUEs	3.22–3.64	13.31–37.92	3.7–9.7	0.50 to 0.87
*QFt.ipk-4A*	2019/BLUEs	4.72–6.23	115.64–149.36	9.1–12.4	0.45 to 0.73
*QFt.ipk-5A*	*ALL*	10.22–31.6	145.01–169.33	29.7–66.4	− 4.15 to − 0.80
*QFt.ipk-5B*	2018	7.16	111.19–116.95	8	1.56
*QFt.ipk-7B.1*	2016/2017/BLUEs	3.25–5.03	0–13.31	5.2–10.1	0.55 to 0.82
*QFt.ipk-7B.2*	2018/2019	3.78–5.99	25.36–37.92	6.5–8.4	0.37 to 1.06

Name of the quantitative trait loci (QTL), environment in which the QTL was identified (Environment), logarithm of odds to declare the presence of a QTL (LOD score), genetic support interval at LOD-1.5 (LOD interval), percentage of phenotypic variance explained by the QTL (*R*^*2*^), estimated effect of putative QTL with positive values for TRI-10703 (parent A) and negative values for TRI-5310 (parent B; additive effect). The full table with description of each individual QTL is available as Supplementary Table S1 online.

### Identification of candidate gene *FT-B1* in the physical region

The mapping onto the IWGSC RefSeq v1.1 of the most significant markers defining the *QTsn.ipk-7B* interval identified for the BLUEs resulted in a physical interval spanning from 6.37 to 21.67 Mb. The region harbored 121 high-confidence genes (Supplementary Table S2). Potential candidates included two genes annotated as CLAVATA3/ESR (CLE)-related protein, six annotated as MADS box transcription factors, and the *FLOWERING* *LOCUS* *T* (*VRN-B3* or *FT-B1*, *TraesCS7B02G013100*) located at 9.7 Mb (9,702,354–9,704,354 bp). Among the 121 high-confidence genes, 26 harbored a total of 56 non-synonymous mutations between both parental lines. All non-synonymous mutations were analyzed with PROVEAN to evaluate their potential effect on the proteins. This analysis identified eight potentially deleterious mutations distributed among six genes including *FT-B1* (Supplementary Table S3). This gene—an ortholog of *Arabidopsis* *FT* and rice *RFT1—*has three predicted exons encoding a phosphatidylethanolamine-binding protein (PEBP). *FT-B1* had a single non-synonymous substitution close to the start codon. At position 19 in the coding sequence, the parental genotype TRI-5310 (parent-B) and the reference Chinese Spring have a G, while TRI-10703 (parent-A) has a C. This mutation modifying an aspartic acid (D) into a histidine (H), although located outside of predicted domains, occurred in a highly conserved position. The 1.5 kb upstream region of *FT-B1* was also searched for transcription factors using the bread wheat epigenomic map (http://bioinfo.sibs.ac.cn/cs_epigenome)^36^. The region harbored potential binding sites for RAP211, ERF2 and RAMOSA1, however, none were polymorphic between both parents.

### Development of a KASP marker significantly associated with TSN

To confirm *FT-B1* as a candidate gene for TSN, a KASP marker was developed for the described mutation in *FT-B1* and genotyping was performed across the whole DH-population. The marker was included in the original matrix for a second round of composite interval mapping for TSN. The KASP marker was the most significant marker for *QTsn.ipk-7B* in the BLUEs and all years but 2017 and explained 10.6–22.9% of phenotypic variance (Supplementary Figure S2, Supplementary Table S4).

### Interaction between *VRN-A1* and *FT-B1* on phenotypic expression of traits

**Figure 4:**
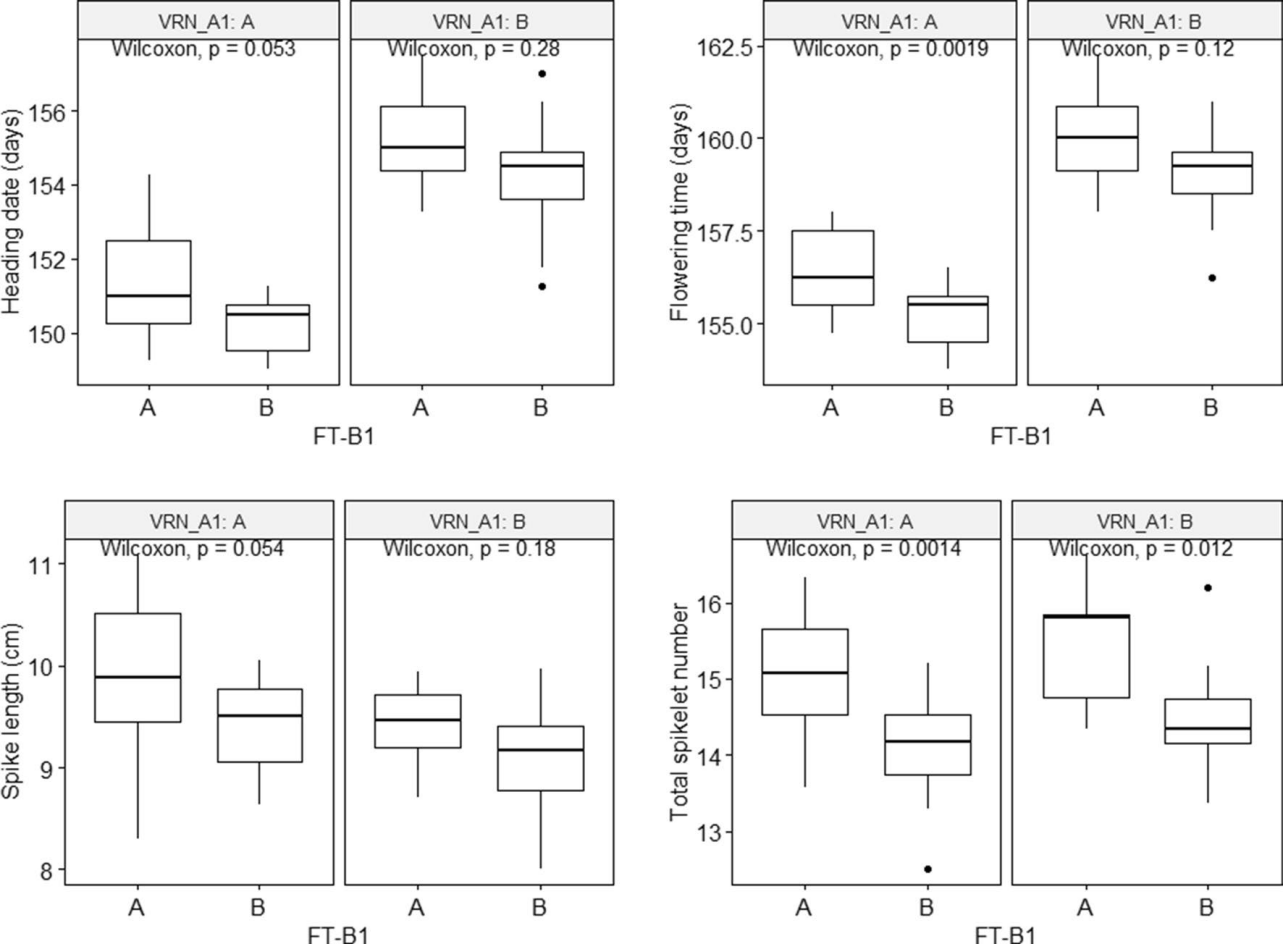
Allele-wise phenotypic distribution of the KASP marker designed for *FT-B1* of the best linear unbiased estimations (BLUEs) for the investigated traits in dominant (**A**) or recessive (**B**) *VRN-A1* background. Significance of the mean differences of the marker alleles obtained from a Wilcoxon rank-sum test.

The genotyping for *VRN-A1* was done according to Zhang et al.^23^ and alleles were coded according to the rest of the data with TRI-10703 as parent A and TRI-5310 as parent B. A two-way factorial ANOVA for the effects of *VRN-A1* and *FT-B1* on all traits revealed a highly significant effect of *FT-B1* but not *VRN-A1* on TSN, while both genes were found significant for the three other traits (Table 3, Supplementary Table S5). The interaction of both genes was not significant. Indeed, individuals carrying the *FT-B1* A allele with amino acid histidine had on average a higher number of spikelets (15.1) than individuals with the B allele (14.3) independent of their *VRN-A1* allele (Fig. 4). Individuals carrying the dominant *Vrn-A1a* allele (A) flowered on average 3 days earlier than individuals with the recessive *vrn-A1* allele (B), while the difference in flowering time caused by the respective *FT-B1* allele was only 1 day. However, this difference was only significant for individuals carrying the dominant *Vrn-A1a* allele (Wilcoxon test, *P* = 0.002). For SL, individuals with the recessive *vrn-A1* allele (B), the spike was on average 0.48 cm (Wilcoxon test, *P* = 0.005) shorter than for the *Vrn-A1a* allele (A).

**Table 3:** *P* values from two-way ANOVAs for the effects of *FT-B1* and *VRN-A1* and their interaction (*FT-B1*:*VRN-A1*) on total spikelet number (TSN), spike length (SL), heading date (HD), and flowering time (FT) based on their best linear unbiased estimations (BLUEs).

	*FT-B1*	*VRN-A1*	*FT-B1*:*VRN-A1*
TSN	***	NS	NS
SL	**	**	NS
HD	***	***	NS
FT	***	***	NS

The full ANOVA results are available as Supplementary Table S5 online. *P < 0.05; **P < 0.01; ***P < 0.001; NS, *P* > 0.05.

### Analysis of frequency of the observed mutation in *FT-B1*

**Figure 5:**
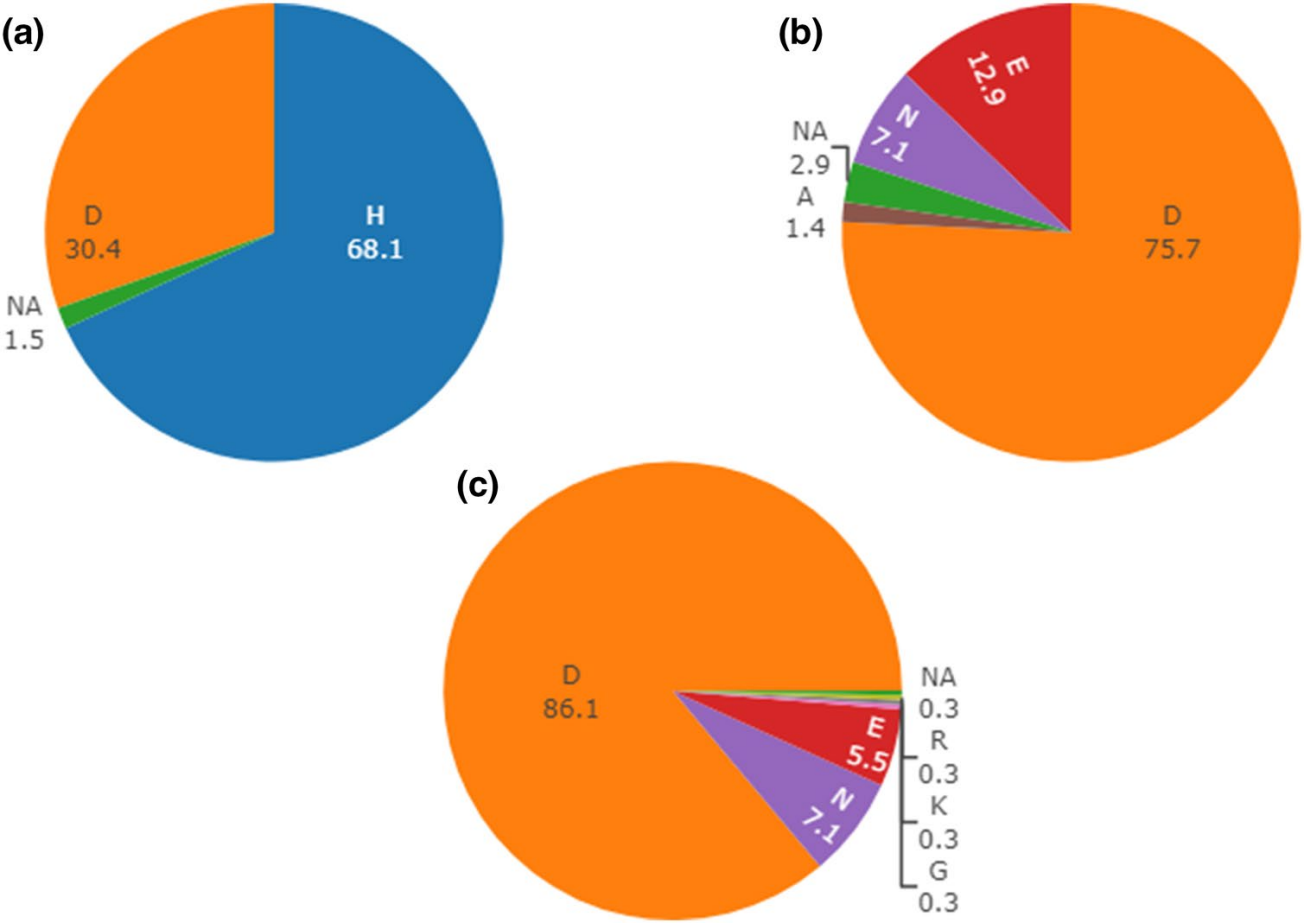
Intraspecific, intragenomic and interspecific diversity for the *FT-B1* mutation. All values are percentages. (**a**) Allele frequency across 135 wheat varieties sequenced with the Axiom marker *AX-94810990* of the nucleotide substitution, with C translating to histidine (H) and G translating to aspartic acid (D). (**b**) Allele frequency for the corresponding amino acid position across 70 PEBP genes annotated in the wheat reference genome. (**c**) Allele frequency for the corresponding amino acid position across 377 homologs retrieved from the PROVEAN analysis across a large diversity of plants.

**Figure 6:**
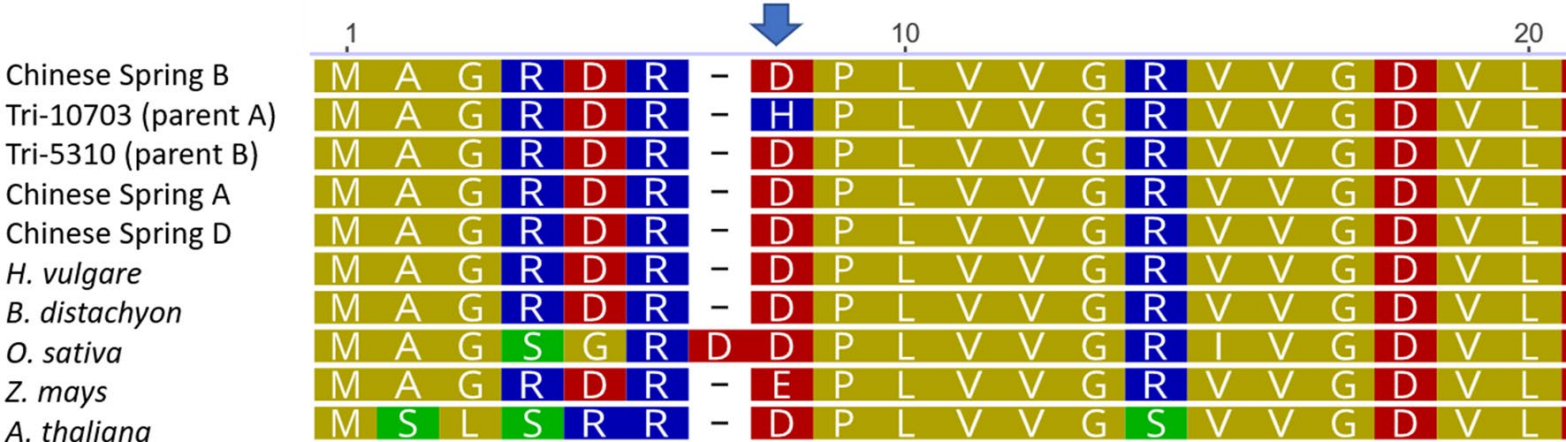
Sequence alignment of the 20 first amino acids of a subset of *FT-B1* homologs including the homoeologues on chromosome 7A and 7D. The wheat reference sequence (Chinese Spring), the sequences of the two parental lines, and *FT-B1* homologs in diverse species viz. *Hordeum* *vulgare*, *Brachypodium* *distachyon*, *Oryza* *sativa*, *Zea* *mays*, and *Arabidopsis* *thaliana*. The blue arrow indicates the position of the aspartic acid (D) to histidine (H) mutation present in the parent A and associated with elevated total spikelet number. The amino acids are color coded according to their side chain polarity with nonpolar (gold), polar (green), basic polar (blue), and acidic polar (red).

In the wheat reference genome, a total of 67 genes, including genes annotated as *MFT*-like and *TFL1*-like, were classified as *FT-B1* paralogs in the EnsemblPlants database with 27 identified in the B genome, whereas, 20 in each of the A- and D-genomes. Among those 70 PEBP genes, including *FT-B1* and its homoeologues on chromosomes 7A and 7D (*TraesCS7A02G115400* and *TraesCS7D02G111600*), the majority possessed an aspartic acid (D) but none had a histidine (Fig. 5b). The homoeologues in the parental lines were identical to each other and to the reference (Fig. 6). In a set of 377 homologs retrieved by PROVEAN from the NCBI database across a large diversity of flowering plants as well as a few Gymnosperms, 86.4% had a D, 7.1% possessed an asparagine (N), and 5.5% had a glutamic acid (E). Three sequences had another amino acid but none had an H (Fig. 5c). This mutation obtained a PROVEAN score of − 4.267 and is therefore considered deleterious. However, in a set of 135 wheat varieties and landraces genotyped with the high-density array Axiom^37^, ca. 68% carried the mutation (marker *AX-94810990*; Fig. 5a) while none of the bread wheat progenitors (eight *T.* *turgidum* and one *Aegilops* *speltoides* accession) included in this genotyping effort had it.

## Discussion

### *FLOWERING LOCUS T* is a likely candidate for a major TSN-QTL

A consistent major QTL for TSN was discovered over 4 years in the field-grown doubled haploid population TRI-10703 × TRI-5310 on chromosome 7BS explaining up to 20.5% of phenotypic variance. The *FLOWERING* *LOCUS* *T* (*FT-B1*) gene located in the most significant QTL interval (7B: 6.3–21.7 Mb) was considered as most likely candidate for the observed effect on TSN, because (1) *FT-B1* carried a non-synonymous mutation causing an amino acid exchange in the parental lines, (2) a QTL analysis including the KASP marker based on the mutation confirmed its significant association with TSN, and (3) *FT-B1* was previously associated with effects on spikelet number in the literature. Moreover, the analysis of all non-synonymous mutations within the QTL interval confirmed *FT-B1* as a likely candidate. Overexpression of *FT-B1* did not have an effect on spikelet number but accelerated flowering time in warm conditions while deletion of *FT-B1* nearly doubled the flowering time and increased spike length and spikelet number^24,25^. According to Boden et al.^38^ the expression of *FT-B1* is regulated by the pseudo-response regulator gene *Ppd-1* and a reduced expression of *FT-B1* was associated with paired spikelet formation and inflorescence architecture.

*FT-B1* is usually associated with flowering time or heading date. Higher *FT-B1* transcripts levels controlled by the insertion of a retrotransposon in the promoter region (“Hope” allele) accelerated flowering time and transgenic winter wheat transformed with the Hope promoter behaved like spring types. The retrotransposon characteristic of the highly expressed *FT-B1* allele from variety “Hope”^22,39^ was not present in our parental lines. Changes in the expression of the wild type *FT-B1* were shown to coincide with major transition of the inflorescence meristem^2^.

### Different effects of *FT-B1* and *VRN-A1* on flowering time and total spikelet number

Our population is segregating for the vernalization genes *VRN-A1* and *VRN-B1*, as well as for the photoperiodism gene *Ppd-D1*. The heading and flowering time were in all years governed by a major QTL on chromosome 5A which represented the effect of *VRN-A1*, while on chromosome 7B representing *FT-B1* only minor QTL were observed for HD and FT (Table 2, Supplementary Table S1). *VRN-A1* had highly significant effects on HD, FT and SL but not on TSN. On the other hand, *FT-B1* was highly significant for TSN and showed small effects on the other three traits (Fig. 4, Table 3). In the correlations, increased TSN was correlated with increased SL as well as with delayed HD and FT. However, *FT-B1* affected spikelet formation without a major effect on the flowering time. It may, therefore, be concluded that the *FT-B1* effects on pre-flowering phases play a major role. The effects of sub-phases in plant growth of wheat on yield and yield components were highlighted by Guo et al.^40,41^, where spikelet initiation and spikelet number was associated with double ridge to terminal spikelet stage. Dixon et al.^24^ reported that flowering time, in contrast to spikelet number, was not responsive to changes in ambient temperature in a *ft-b1* NIL, suggesting that the promotion of inflorescence development between the double ridge and terminal spikelet stages is regulated by *FT*-independent thermally responsive factors. In contrast to *Arabidopsis*, where *FT* induces flowering at elevated growth temperature^42^, the accelerated development pace of domesticated wheat under high temperature is largely independent of *FT*^24,43^. Li et al.^44^ investigated the interactions between the three wheat MADS-box genes *VRN1*, *FUL2* and *FUL3* and showed delayed formation of the terminal spikelet and increased number of spikelets per spike in *VRN1-null* and *ful2-null* mutants. Flowering delays in *VRN1-null* mutants were associated with reduced *FT1* transcript levels in leaves. Also changes in expression level of *FLOWERING* *LOCUS* *T2* (*FT2*), the closest paralog of the *FT1* gene in temperate grasses, were associated with changes in spikelet number, flowering time and fertility^45,46^. Another QTL-study reported three pleiotropic QTL regions associated with spikelet number and heading date on chromosomes 2A, 7A and 7D, with *FT-A1* considered as candidate gene for *QTspn/Hd.cau-7A*^9^.

### A non-synonymous mutation in exon1 of *FT-B1* is widespread in the species *T. aestivum*

Sequencing of the parental lines of the DH-population allowed us to screen for mutations and indels affecting the genes annotated in the physical interval corresponding to the major QTL. Evaluating all non-synonymous mutations affecting the 121 high-confidence genes present in the QTL interval, only eight were considered as potentially deleterious. Among those, we identified a non-synonymous substitution in exon1 of *FT-B1* in our parental lines changing the aspartic acid (D) into a histidine (H). Analyzing a collection of more than 300 sequences of *FT-B1* homologs, including homoeologues and paralogs, covering a large diversity of land plants revealed that the mutation was novel in hexaploid wheat. However, it appeared that the mutation, present with the array code *AX-94810990* on the high-density genotyping arrays Axiom 820 K and 35 K, is probably widespread (68%) in wheat varieties and landraces but absent in the few tetraploid and *Ae.* *speltoides* accessions screened to validate the 820 K array^37^. The mutation from D to H confers a phenotype similar but weaker than the complete loss of *FT-B1* (and 400 other genes) described in Dixon et al.^24^ and Finnegan et al.^25^. We conclude that this could be a mutation potentially only slightly impairing the protein which remains suitable in breeding. Taken together, our results suggest that this variant is a novelty private to hexaploid wheat where it was favored potentially for its positive impact on the grain yield. It may also have been selected for winter hardiness as the histidine mutation was found to be associated with an increased frost tolerance^47^. Interestingly, analysis of transgenic rice showed that *Hd3a*—a rice *FT* homolog—accumulated in axillary meristems and promoted the formation of branches^21^. Analyzing *FT*-like genes in maize and rice, Zheng et al.^34^ have shown that those genes had been under positive selection during domestication allowing the plants to adapt to different environments during the range expansion of by modulating their flowering time.

## Conclusion

Linkage mapping revealed the effects of *FT-B1* on total spikelet number in the genetic background of a doubled haploid spring wheat population, while the heading and flowering time was dominated by the effects of a segregating *VRN-A1* gene. A non-synonymous mutation in *FT-B1* causing an amino acid exchange from aspartic acid to histidine was observed in the parental lines of the mapping population. The observed mutation is widespread in hexaploid wheat, but could not be detected in any other orthologs and paralogs within the higher plants. It may be speculated that effects of *FT-B1* on subphase duration of wheat affect the final spikelet number rather than the flowering time. This is a first report about a structural variation in *FT-B1* influencing TSN, which is also inheritable, while other reports were mainly based on expression analysis or the complete loss described in Dixon et al.^24^ and Finnegan et al.^25^ consisting of the first 23 Mb of chromosome 7B encompassing more than 400 genes.

## Materials and methods

### Plant material and field trials

The doubled haploid (DH) spring wheat population-2 consisting of 159 lines was previously described in Muqaddasi et al.^35^. The parental lines, conserved at the IPK Genebank, were TRI-10703 (landrace from Greece) and TRI-5310 (variety Eureke from France). The accessions were originally selected for their contrasting anther extrusion phenotypes but exhibited a large difference for total spikelet number per spike (TSN). The DH population and its parental lines were sown at the end of March and grown in 2 m^2^ plots on the field in IPK Gatersleben for four consecutive cropping seasons (2016–2019) and characterized each year for TSN, spike length (SL), heading date (HD), and flowering time (FT).

### Phenotyping and data analysis

The spike traits (TSN and SL) were recorded from ten spikes per plot as the total number of spikelets and spike length in centimeters (cm) from basal spikelet to the top of a spike but excluding the awns. The flowering traits (HD and FT) were recorded for each year as the number of days between the first of January and when approximately half of the spikes in a plot emerged and flowered, respectively.

Phenotypic data were analyzed following Muqaddasi et al.^35^. Specifically, best linear unbiased estimations (BLUEs) were calculated per year for the spike traits and across years for all traits by assuming fixed effects for the intercept and the genotype and all others as random. All phenotyping data analyses, including ANOVAs, Pearson’s correlation coefficient analyses, repeatability among replicates, broad-sense-heritability across years, and statistical tests, were performed using the software R^48^.

### Genotyping and QTL mapping

The 159 lines were genotyped with a custom-designed 15 k Infinium array^49^ by the company TraitGenetics GmbH in Gatersleben, Germany, that resulted in 3,457 polymorphic markers. The 15 k Infinium array was created as a cost-effective option based on the 90 k Infinium array described by Wang et al.^50^. Data filtering and linkage map construction were previously described in Muqaddasi et al.^35^.

The quantitative trait loci (QTL) underlying the four traits were analyzed by composite interval mapping (CIM)^51^ in Windows QTL Cartographer 2.5^52^. For each trait, single year and across year BLUEs were used to calculate both forward and backward regression. The step size was set at 2 cM. To identify significant QTL at α = 0.05, thresholds for the logarithm of odds (LOD) values were estimated with 1000 permutation^53^. Confidence interval for the significant QTL were defined as a continuous genomic region comprised within a 1.5-LOD from the marker with the highest LOD per chromosome passing the threshold^54^. QTL repeatedly identified in the same interval each year and in their BLUEs were defined as major and consistent.

### Analysis of the physical region

All genomic analyses were performed in Geneious Prime 2020.0.5 (https://www.geneious.com). First the sequences of the markers corresponding to the consistent QTL’s genetic intervals were obtained from Wang et al.^50^ and BLASTed on their assigned chromosome of the wheat genome reference sequence^55^ to define genomic intervals. The physical interval defined for *QTsn.ipk-7B* was retrieved from the assemblies of the parental lines sequenced at 10 × coverage with Illumina paired-end sequencing (HiseqXTEN PE150) and assembled as described in Muqaddasi et al.^35^. The sequences of the genes present in the interval were mapped to the sequences of the parental lines and then extracted. Mapping was performed with the Geneious mapper set to “Custom sensitivity” based on the “Medium–Low Sensitivity” parameters but with a maximum gap size of 2000 bp to accommodate for missing data regions. Multiple sequence alignments (MSA) for each gene consisting of the sequences of the reference and of the two parental lines were obtained by running the same mapping procedure using the genic sequences of Chinese Spring as reference. Polymorphisms between both parental lines were annotated in the MSAs. To narrow down to potential candidates, the functional annotations of the genes in the interval were inspected which revealed the presence of two CLAVATA3/ESR (CLE)-related proteins, six MADS box transcription factors, and the *FLOWERING* *LOCUS* *T* (*VRN-B3* or *FT-B1*, *TraesCS7B02G013100*). The potential effect of the non-synonymous substitutions affecting *FT-B1* and all other high-confidence genes present within the interval were analyzed with PROVEAN (Protein variation effect analyzer)^56^. The bread wheat epigenomic map (http://bioinfo.sibs.ac.cn/cs_epigenome)^36^ was used to identify the location of potential transcription factors in the 1.5 kb region upstream from *FT-B1*.

### Interaction between *FT-B1* and *VRN-A1*

To test the potential interaction between the candidate gene *FT-B1* and the vernalization gene *VRN-A1*, the whole population was genotyped with a Kompetitive Allele Specific PCR (KASP) marker based on the *FT-B1* SNP (Supplementary Table S6) and *VRN-A1* primers^23^. All the traits were analyzed with a two-way factorial ANOVA and allele-wise phenotypic distribution was illustrated by plotting the boxplots. The effect of the different alleles at both loci for all traits were evaluated with a Wilcoxon rank-sum test.

### Intragenomic, intra- and interspecific analyses

The EnsemblPlant database (https://plants.ensembl.org) was used to identify and recover the protein sequences of all genes classified as paralogs of the three homoeologues (*TraesCS7A02G115400*, *TraesCS7B02G013100*, and *TraesCS7D02G111600*). The sequences were then aligned with MAFFT v7.450^57,58^ in Geneious. The CerealsDB database (www.cerealsdb.uk.net)^59^ and the function “Extract genotype for a specific SNP” were used to obtain the genotyping score of the Axiom 820 K array marker *AX-94810990* covering the non-synonymous mutation identified in *FT-B1*. Samples with the ancestral (G) or the derived allele (C) were scored with BB and AA, respectively. Among the 475 genotypes recovered, only the samples identified as varieties, landraces, tetraploid and *Aegilops* *speltoides*, according to Winfield et al.^37^, were kept to evaluate the frequency of the mutation across bread wheat and its progenitors. Interspecific analyses were performed on the set of 377 homolog sequences retrieved by the PROVEAN analysis.

## Supplementary Information

Supplementary Information 1.

Supplementary Information 2.
