# Supplementary material for: Linkage mapping identifies a non-synonymous mutation in FLOWERINGLOCUST (FT-B1) increasing spikelet number per spike
Source: Sci Rep. 2021 Jan 15;11:1585. doi: 10.1038/s41598-020-80473-0 (PMC7811022; doi:10.1038/s41598-020-80473-0)
Supplement: Supplementary file 1 — Supplementary Information 1. [file 41598_2020_80473_MOESM1_ESM.docx]

**Linkage mapping identifies a non-synonymous mutation in *FLOWERING LOCUS T* (*FT-B1*) increasing spikelet number per spike**

Jonathan Brassac*, Quddoos H. Muqaddasi, Jörg Plieske, Martin W. Ganal, and Marion S. Röder

* Corresponding author: Jonathan Brassac ([brassac@ipk-gatersleben.de](mailto:brassac@ipk-gatersleben.de))

**Supplementary Figures**

**
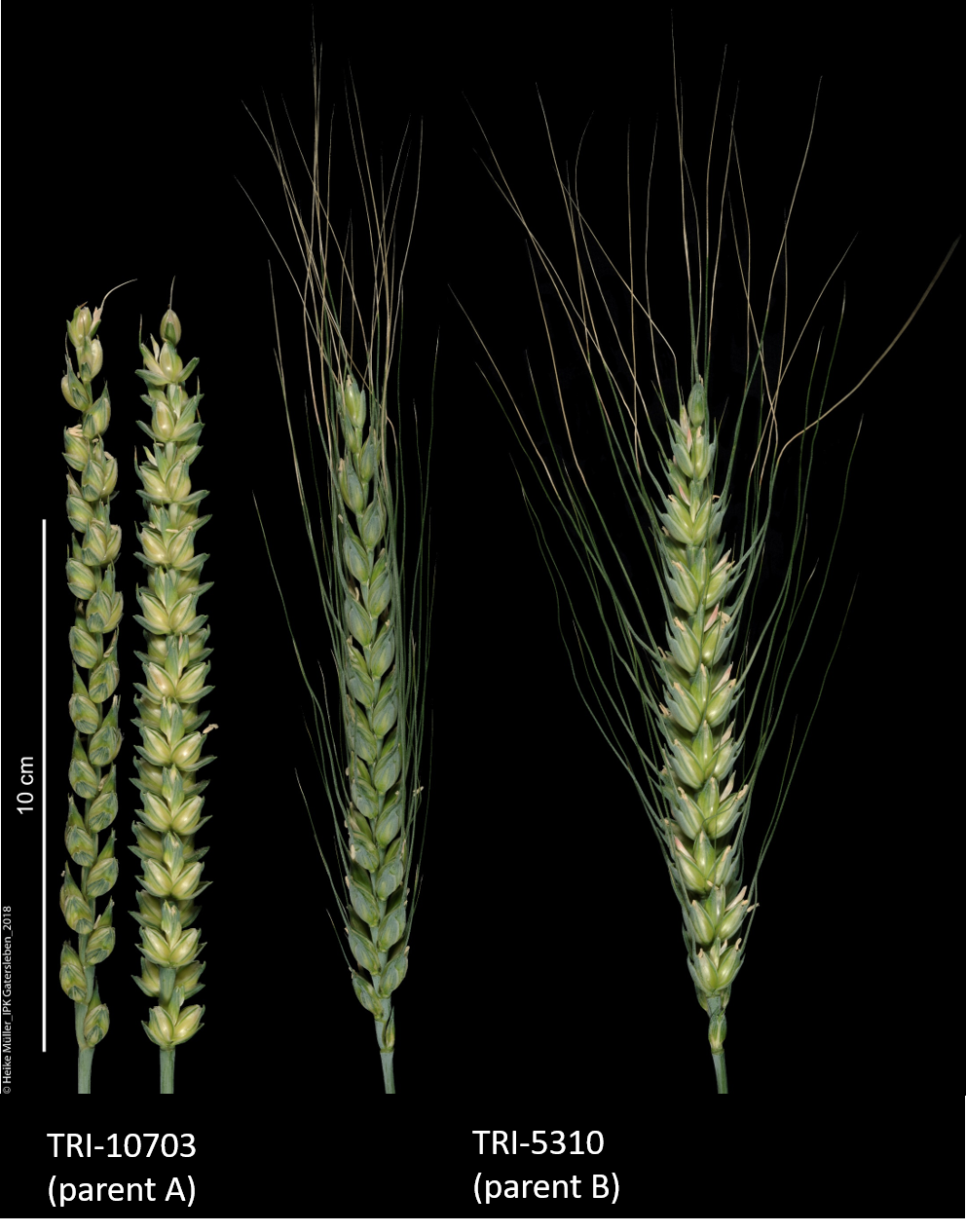
**

**Figure S1.** Spike morphology of parental lines TRI-10703 (left) and TRI-5310 (right) with front view and side view for each spike.

**
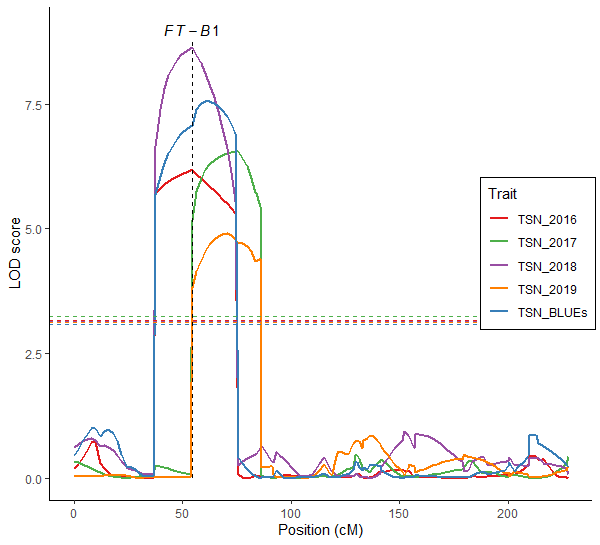
**

**Figure S2** Quantitative trait loci (QTL) map of total spikelet number (TSN)on chromosome 7B in a DH-population of spring wheat grown over four years and their corresponding best linear unbiased estimations (BLUEs). The horizontal dashed lines indicate the corresponding logarithm of odds (LOD) threshold estimated at α = 0.05 with 1000 permutations to assess the QTL significance. The position of the KASP marker developed for the mutation described in *FT-B1* is indicated with a vertical dashed line.
